# Editorial: Palliative Care in Neurology

**DOI:** 10.3389/fneur.2019.01370

**Published:** 2020-01-09

**Authors:** David Oliver, Marianne de Visser, Raymond Voltz

**Affiliations:** ^1^Tizard Centre, University of Kent, Canterbury, United Kingdom; ^2^Amsterdam University Medical Centre, Amsterdam, Netherlands; ^3^Department of Palliative Medicine, Faculty of Medicine and University Hospital, University of Cologne, Köln, Germany

**Keywords:** palliative care, neurology, collaboration, ethical issues, quality of life

The role of palliative care for people with progressive neurological disease has been increasingly discussed over the last 20 years (1–3). Initially, the focus was on amyotrophic lateral sclerosis (ALS) which “represents a paradigm for palliative care in neurological diseases” (4). Indeed, progression in ALS is rapid, leading to severe disability, rendering the patients fully dependent on the support of carers, and death occurs ~3 years after disease onset in half of the patients. More recently a palliative care approach also found its way to diseases such as high-grade glioma of which the course is also relentlessly progressive like in ALS, Parkinson's disease (PD), and multiple sclerosis (MS). The two latter are also associated with progressive disability and a shortened life expectancy but have a more prolonged and thus unpredictable course.

The European Academy of Neurology (EAN), in collaboration with the European Association for Palliative Care (EAPC) have produced a Consensus paper on neurological palliative care, which outlines the need for a wider assessment of patients—physical, psychological, social and spiritual, and including consideration of end of life care and discussion of hastened death (3). Moreover, the support of carers, both family and professional has been emphasized (3).

This Research Topic has aimed to look at new developments in the palliative care of patients with neurological disease and the editors were heartened by the response and the papers submitted. They consider many different aspects of care and several different disease groups.

The need to assess carefully the various symptoms of all patients is emphasized in the paper by Anneser et al.. They found that neurological symptoms were common, both in patients with neurological diseases and other patients receiving palliative care. These symptoms may affect the quality of life of patients. However, the survey of neurologists in the Netherlands (Walter et al.) showed that discussions about treatment restrictions and the consideration of palliative care in PD and MS were often delayed until the later stages of the disease progression—cognitive decline was often the trigger. This has again shown that education of neurologists is important in enabling discussion about deterioration and end of life to take place earlier in the disease progression, as was suggested by the EAN/EAPC paper (3).

One way of enabling professionals to become more aware of the prognosis of the patient may be the use of the “Surprise Question”—“Would you be surprised if your patient would die in the next 12 months?” This was found to be useful, particularly when combined with an assessment of the symptom burden (Ebke et al.). There is also a need to ensure that the necessary expertise in the management of palliative care issues for neurological patients is more widely available. The innovative use of telemedicine in Bavaria, Germany was shown to help and support palliative care teams in the management of patients with neurological disease, when they do not have the specific expertise required (Weck et al.).

The role of palliative care for patients with ALS has been established for many years (5). The physical aspects may be complex and in particular the use of non-invasive ventilation (NIV) for patients with respiratory muscle weakness was shown to be associated with improved QoL and survival (6), albeit many questions remained about timing and the minimum forced vital capacity (FVC). Khamankar et al. present a new approach to commencement of NIV with a protocol suggesting initiation for patients with a FVC of 80% or less, a higher level than is usually suggested, together with encouragement for regular usage and cough assistance. This approach would seem to improve survival-−30.8 months in their cohort, compared to a standard regime. This is a challenge to look carefully at how NIV is discussed and used.

The psychosocial issues for people with ALS are profound, facing a progressive deterioration in physical function over a short period of 2–3 years. Grabler et al. have shown that although depression, anxiety, and death anxiety are not particularly common in ALS patients, they found that they widely correlate with each other and should be addressed altogether. Caregivers' strain was related to both depression and anxiety. This again shows the importance of the care and support, and often intervention, for caregivers, to help them care for patients with ALS. A group in Leipzig in Germany are developing a short-term intervention “Managing Cancer and Living Meaningfully (CALM)” which has been adapted for patients with ALS (Oberstadt et al.). This now needs to be validated to ascertain if this approach would be helpful in allowing patients to receive the support they need, both in terms of symptoms, relationships, and psychosocial well-being. The use of high technology alternative and augmentative communication systems is often very important as communication becomes difficult and Linse et al. discuss the issues of ensuring that equipment is used effectively to help patients strengthen self-determination, improve quality of life and reduce caregiver burden.

There is increasing interest in the role of palliative care for people with Parkinson's disease. The paper from Hanover, Germany shows that the quality of life of people with PD is poor, with motor deficits, impairment of daily living, depression and cognitive loss (Klietz et al.). However, although 72% had an unmet palliative care need, only 2.6% had received palliative care input. A new approach is described by Eggers et al. where in Cologne, Germany a network has been developed, with a model of care provided by a movement disorder neurologist and a PD nurse collaborating with neurologists across the area. This service did not seem to see late-stage disease patients and there appeared to be poor access and loss of follow-up toward the end of life. Many PD patients may be admitted for nursing home care as the disease progresses. Lex et al. looked at residents who are in a late stage of Parkinson Disease in residential care in Salzburg, Austria, and found that despite their severe disease, limiting their activities and mobility, they did not have a significant symptom burden and were content with their quality of life. They appreciated the closeness of family and nursing home care and family members were often reassured that the resident was being cared for and their anxiety and burden had lessened.

van der Steen et al. have shown that people with PD and those with dementia face uncertainty and increasing disability and cognitive loss and that palliative care can be helpful. There is a need to develop a palliative care approach to cope with the variable and protracted deterioration, ensuring symptoms are managed effectively, carers are supported and advance care plans are considered earlier.

Although in the past palliative care for neurological patients has tended to focus on ALS, and to a lesser extent PD and MS, there is increasing awareness of the role for other disease groups. Stroke is a very common cause of disability and death across the World and there is a need to look at this patient group and their palliative care needs (7). Steigleder et al. consider the challenges of providing care for stroke, when the outcome is uncertain and there may be rapid changes. They argue for a wider consideration of palliative care and consider the barriers of the implementation of this, and look at possible ways forward. Patients with high-grade glioma also face an uncertain future and the paper from Renovanz et al. shows that both patients with glioma and their carers have many psychological issues, particularly when receiving chemotherapy. They press for greater support of both patients and carers at these difficult times.

Many patients receiving palliative care may have neurological symptoms which need to be assessed and addressed. Grönheit et al. discuss the difficult area of managing epileptic seizures and status epilepticus and provide practical advice on medication and the mode of administration. Restless legs can be a very distressing symptom, for both person and bed partner. Gärtner et al. provide an interesting insight with a case study of a patient who responded to morphine. There is still much to learn and case studies may give us an insight, which can in turn lead to a deeper understanding and more effective management of symptoms.

The papers in this volume provide an opportunity to look at palliative care for neurological patients. This is expanding and in the USA the concept of neuro-palliative care is increasing—neurologists with extra palliative care training and experience, who are able to develop the multidisciplinary approach to people with neurological diseases (8). There are many challenges and all involved in the care of patient with neurological disease—neurologists, rehabilitation physicians, the wider multidisciplinary teams, palliative care specialists, primary care teams, patients and families—need to face these issues, and may require training to cope with the new areas (3). However, with the increase in a generalist palliative care approach—listening to patients, helping to set goals and assessing all areas of care (physical, psychological, social, and spiritual)—and collaborating with specialist palliative care services for more complex areas we can all learn to help patients with neurological diseases, to maintain their quality of life and enable a better quality of death (9, 10).

## Author Contributions

All authors listed have made a substantial, direct and intellectual contribution to the work, and approved it for publication.

### Conflict of Interest

The authors declare that the research was conducted in the absence of any commercial or financial relationships that could be construed as a potential conflict of interest.
